# Network Discovery Pipeline Elucidates Conserved Time-of-Day–Specific cis-Regulatory Modules

**DOI:** 10.1371/journal.pgen.0040014

**Published:** 2008-02-01

**Authors:** Todd P Michael, Todd C Mockler, Ghislain Breton, Connor McEntee, Amanda Byer, Jonathan D Trout, Samuel P Hazen, Rongkun Shen, Henry D Priest, Christopher M Sullivan, Scott A Givan, Marcelo Yanovsky, Fangxin Hong, Steve A Kay, Joanne Chory

**Affiliations:** 1 Plant Biology Laboratory, The Salk Institute for Biological Studies, La Jolla, California, United States of America; 2 Department of Botany and Plant Pathology, Oregon State University, Corvallis, Oregon, United States of America; 3 Center for Genome Research and Biocomputing, Oregon State University, Corvallis, Oregon, United States of America; 4 Section of Cell and Developmental Biology, University of California San Diego, La Jolla, California, United States of America; 5 Howard Hughes Medical Institute, The Salk Institute for Biological Studies, La Jolla, California, United States of America; Howard Hughes Medical Institute, Northwestern University, United States of America

## Abstract

Correct daily phasing of transcription confers an adaptive advantage to almost all organisms, including higher plants. In this study, we describe a hypothesis-driven network discovery pipeline that identifies biologically relevant patterns in genome-scale data. To demonstrate its utility, we analyzed a comprehensive matrix of time courses interrogating the nuclear transcriptome of Arabidopsis thaliana plants grown under different thermocycles, photocycles, and circadian conditions. We show that 89% of *Arabidopsis* transcripts cycle in at least one condition and that most genes have peak expression at a particular time of day, which shifts depending on the environment. Thermocycles alone can drive at least half of all transcripts critical for synchronizing internal processes such as cell cycle and protein synthesis. We identified at least three distinct transcription modules controlling phase-specific expression, including a new midnight specific module, PBX/TBX/SBX. We validated the network discovery pipeline, as well as the midnight specific module, by demonstrating that the PBX element was sufficient to drive diurnal and circadian condition-dependent expression. Moreover, we show that the three transcription modules are conserved across *Arabidopsis*, poplar, and rice. These results confirm the complex interplay between thermocycles, photocycles, and the circadian clock on the daily transcription program, and provide a comprehensive view of the conserved genomic targets for a transcriptional network key to successful adaptation.

## Author Summary

As the earth rotates, environmental conditions oscillate between illuminated warm days and dark cool nights. Plants have adapted to these changes by timing physiological processes to specific times of the day or night. Light and temperature signaling and the circadian clock regulate this adaptive response. To determine the contributions of each of these factors on gene regulation, we analyzed microarray time course experiments interrogating light, temperature, and circadian conditions. We discovered that almost all *Arabidopsis* genes cycle in at least one condition. From a signaling perspective, this suggests that light, temperature, and circadian clock play an important role in modulating many physiological pathways. To clarify the contribution of transcriptional regulation on this process, we mined the promoters of cycling genes to identify DNA elements associated with expression at specific times of day. This confirmed the importance of several DNA motifs such as the G-box and the evening element in the regulation of gene expression by light and the circadian clock, but also facilitated the discovery of new elements linked to a novel midnight regulatory module. Identification of orthologous promoter elements in rice and poplar revealed a conserved transcriptional regulatory network that allows global adaptation to the ever-changing daily environment.

## Introduction

The circadian clock functions to optimize physiology and metabolism to the correct time of the day and is crucial for fitness. Organisms experience the external environment as a dynamic relationship between daily changes in temperature (thermocycles) and light (photocycles) that vary by season and latitude (Figures S1 and S2). Consequently, most species have evolved an endogenous circadian clock with a period of about 24 h that ensures internal biological processes are appropriately synchronized with the daily changes in the environment [1–3]. Together, environmental cycles and the circadian clock phase gene expression, metabolism, and physiology to the correct time of the day [4].

While much is known about how organisms sense light and integrate photocycles to synchronize the circadian clock, little is known of how ambient thermocycles are sensed and integrated. However, the effect of thermocycles on the circadian clock has been described in multiple model systems [5–7]. Thermocycles of only a few degrees are sufficient to set the phase of the circadian clock in most organisms, and recent data suggest that organisms sense thermocycles directly through the circadian clock [8–10]. In *Arabidopsis thaliana,* thermocycles of 10 °C difference are dominant over photocycles for setting the phase of gene expression, consistent with the notion that multiple forms of the circadian oscillator may exist that have temperature- and light-specificity [7]. How and to what extent ambient thermocycles influence daily transcription remains unknown.

Diurnal conditions and the circadian clock regulate a wide variety of downstream events in higher plants. Microarray time course data predicted that between 6% and 15% of the *Arabidopsis* transcriptome is regulated by the circadian clock [11,12], while an enhancer trap study estimated that 36% of the transcriptome was regulated by the circadian clock [13]. In comparison, it was estimated that 30% to 50% of the transcriptome cycled under the diurnal conditions of photocycles and continuous temperature [14]. Under both diurnal and circadian conditions, transcript abundance is phased to every hour over the day, and this regulation forms the foundation for time-of-day–specific biological activities [14,15]. Previous studies identified several promoter elements involved in phase-specific light or circadian regulation of gene expression in plants [13,15–17]; however, it is still unclear how a transcriptional network is constructed that orchestrates multiple modes of phase-specific expression over the day. Oscillatory behaviors/patterns provide multiple levels of redundancy, thereby making analysis of circadian-regulated genes an ideal system to dissect complex networks.

Mining biologically relevant information from large datasets is a current focus of major research efforts. Multiple methodologies such as clustering have been developed to organize and infer the patterns emerging from microarray data. Conventional microarray clustering approaches are based on dividing the input data into related subsets based on a distance metric (Hierarchical, K-Means, Self Organizing Maps, Support Vector Machines), or principal-component analysis. Regardless of the clustering method used, groups of co-expressed genes may be co-regulated to form the foundation for analyses of promoter sequences to identify important cis-regulatory elements. Using these methodologies, it has been possible to predict gene regulatory networks from expression profiling in yeast [18,19]; however, larger and more complex eukaryotic systems will require new conceptual frameworks to unravel transcriptional networks.

In this study we describe a network discovery pipeline, which is a conceptual framework that utilizes predefined hypotheses to search for biologically relevant patterns at multiple levels of genome-scale data. Using this pipeline, we analyzed eleven diurnal and circadian time courses in the reference plant *Arabidopsis*. We demonstrate that the pipeline is successful at defining conserved cis-regulatory modules involved in phase-specific expression underlying the diurnal and circadian transcriptional network.

## Results

### Photocycles, Thermocycles, and the Circadian Clock Drive Time-of-Day–Specific Transcript Abundance

**Figure 1:**
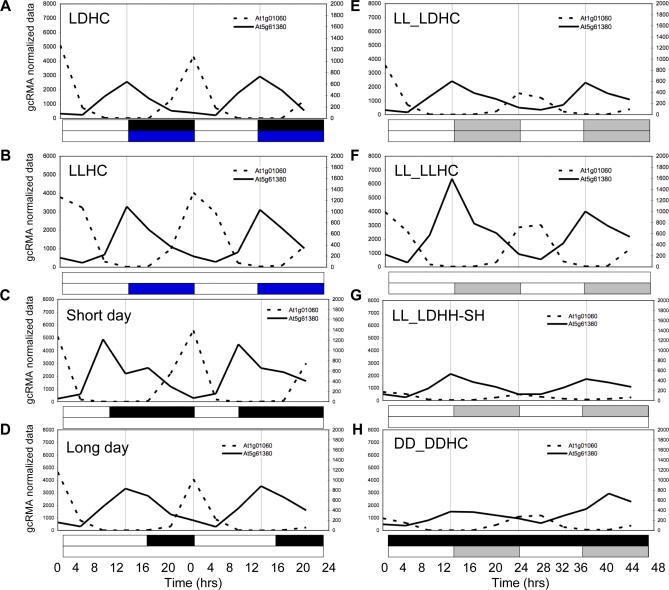
*LHY* (At1g01060) and *TOC1* (At5g61380) Cycle as Expected across Diurnal and Circadian Time Courses Unlogged gcRMA normalized time course data are plotted as a function of time in hours. Light and temperature conditions are indicated below the data. In the diurnal conditions (A–D), the upper condition box represents photocycles; the black boxes indicate the dark period. The lower condition box represents thermocycles with blue boxes indicating 12 °C and the open boxes 22 °C. Specifics for each condition are outlined in Table 1 and Figure S3. Grey boxes represent subjective night or cold temperatures under circadian conditions. (A) LDHC, (B) LLHC, (C) Long day, (D) Short day, (E) LL_LDHC, (F) LL_LLHC, (G) LL_LDHH, and (H) DD_DDHC.

In nature, photocycles and thermocycles provide daily cues that set or entrain the circadian clock, regulating a diverse set of biological functions [20]. To understand how photocycles, thermocycles, and the circadian clock interact to control time-of-day–specific transcript abundance in *Arabidopsis*, we analyzed eleven two-day time courses comprising 132 Affymetrix microarrays (Figure S3). For seven of the time courses, 7-d-old seedlings were sampled every 4 h over 2 d under thermocycles (HC, hot/cold) and/or photocycles (LD, light/dark), or continuous conditions (LL, continuous light; DD, continuous dark, HH, continuous hot; Table 1, Figure 1, and Figure S3). The four remaining time courses LDHH-ST (Stitt), LDHH-SM (Smith), LL_LDHH-SH (Harmer), and LL_LDHH-AM (Millar), were described previously [12,14,21,22] and differ in sampling strategies (Text S1).

**Table 1:**
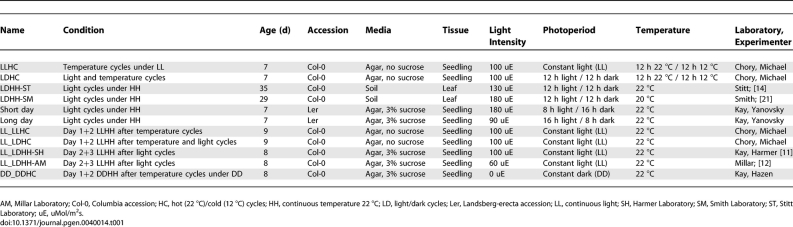
Sampling Strategy for Diurnal and Circadian Time Courses

In this study, we utilized thermocycles of 12 h at 22 °C (hot) and 12 h at 12 °C (cold) for three reasons: (1) the circadian clock is temperature-compensated between 22 °C and 12 °C, which means that the period will remain relatively constant between these temperatures [23]; (2) these thermocycles drive a temperature-sensitive oscillator [7]; and (3) these conditions represent the average environmental changes across latitude and season for Arabidopsis thaliana in its natural habitat [24]. The thermocycles and photocycles were not intended to exactly replicate natural conditions. Rather, they were designed to build on widely used conditions utilized by the *Arabidopsis* community. It has been suggested that in nature thermocycles are shifted 6 h later than photocycles [25]. However, real time temperature and light data recorded over multiple years shows that temperature always increases linearly with light at the beginning of the day and depending on season, microclimate, and latitude, decreases more slowly than light at the end of the day (Figures S1 and S2). Based on these climatic data, we decided to superimpose thermocycles and photocycles for the LDHC time course, and refer to both the cold/hot and dark/light transition as time zero (start of the day).

To determine the quality of the time-course data, we visually inspected the expression patterns of known circadian clock genes [11,12,14,21] and verified the microarray expression pattern for a subset of these genes by qPCR using independent replicates. Figure 1 illustrates the expression pattern of two core circadian clock genes, *LATE ELONGATED HYPOCOTYL* (*LHY)* and *TIMING OF CAB1* (*TOC1),* across eight of the conditions. The expression pattern of *LHY* and *TOC1* highlight key trends across conditions. First, the time of peak transcript abundance (phase) of *LHY* and *TOC1* relative to our definition of time zero (dawn) is the same across all conditions (Figure 1). This result is important because it demonstrates that defining the cold/hot and dark/light transition as time zero provides an accurate reference point to compare phase between datasets. Second, since we normalized all the time courses together, we were able to compare absolute expression levels across conditions (Material and Methods) and found that the light exposure time was almost always positively correlated with expression level (the highest were under thermocycles alone, which is continuous light with temperature synchronizing the cells). Third, photoperiod shifts the phase of some (e.g., *TOC1*), but not all, core circadian clock transcripts (*LHY*; Figure 1C versus 1D). All diurnal and circadian data can be searched, graphed, and downloaded at the DIURNAL web site (http://diurnal.cgrb.oregonstate.edu/) [26].

### Network Discovery Pipeline

We developed a hypothesis-driven bioinformatics platform that can be intuitively applied to all sorts of large-scale data. Our pipeline hinges on two linked concepts: organizing data in a conceptual “series” and defining biologically relevant “patterns” within that series. Serializing data establishes innate contrasts in the data that can then be searched based on predefined hypotheses. Our method is designed to reduce the search space and identify only the biologically relevant information. While our platform may miss unanticipated patterns, both type I and II errors (false positives and false negatives) are reduced by applying predefined patterns (hypotheses) at multiple steps.

**Figure 2:**
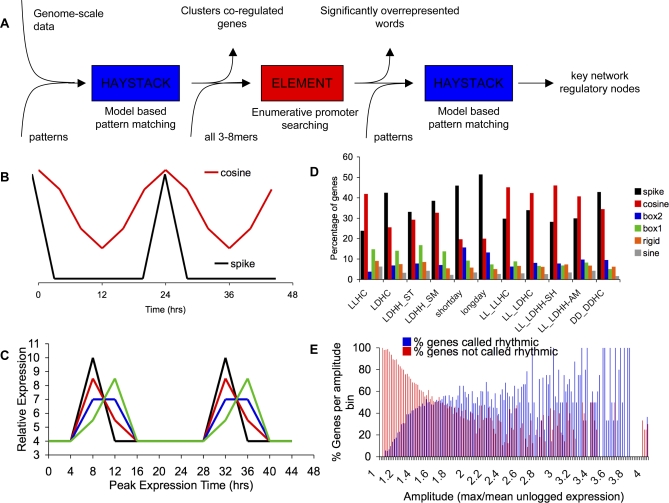
The Network Discovery Pipeline Identifies Patterns in Large Datasets (A) Data flow of the network discovery pipeline. (B) Models used to identify diurnal and circadian regulated transcripts: Spike and Cosine. (C) Models shifted by 1-h increments to quantify time-of-transcript abundance. All models were shifted in 1-h increments, causing a change in the model form and shape over the day to account for changing waveforms caused by our 4-h sampling strategy. The models are presented in batches covering 4-h time intervals for presentation purposes: the spike model shifted to Zeitgeber Time 08 (ZT08, 8 h after dawn, black line), ZT09 (red line), ZT10 (blue line), and ZT11 (green line). (D) Model usage broken down by percent of genes called rhythmic by each model. Spike (black), cosine (red), box2 (blue), box1 (green), rigid (orange), and sine (grey) models were used to identify cycling transcripts. The model with the highest correlation was retained and then used to predict cycling if it had a significant correlation (r ∼ 0.8, FDR ≤ 5.8%). (E) Comparison of percent of genes called rhythmic versus genes not called rhythmic by amplitude. Amplitude was estimated by dividing the maximum by the mean expression value across the time course. Genes were then plotted as the percentage that have specific amplitude and are either rhythmic (blue) or arrhythmic (red).

The analysis starts (Figure 2A) by arranging each microarray dataset into a 48-hour (12 time point) series and searching for specific patterns of expression using HAYSTACK, a model-based pattern-matching algorithm. The resulting lists of co-regulated genes are then used to seed an enumerative promoter-searching tool called ELEMENT, which generates a significance statistic for every potential cis-regulatory word (3–8mer). Finally, the significance statistics for all potential cis-regulatory words are serialized and searched using HAYSTACK to reveal co-occurring elements that form the basis of transcriptional network modules. The components of the pipeline are available at http://haystack.cgrb.oregonstate.edu/ and http://element.cgrb.oregonstate.edu/.

HAYSTACK is designed to find rare occurrences of very specific patterns in a large dataset and provides an alternative method for clustering microarray data by grouping genes whose expression patterns match the same or similar HAYSTACK patterns. The Web version of HAYSTACK can be used to compare any large-scale dataset representing at least three data points (e.g., treatments, genotypes, time points) against a set of user-supplied model patterns. Here, we have focused on time course data. We developed multiple cycling patterns based on diurnal and circadian time course studies available in the literature: asymmetric, rigid, spike, cosine, sine, and/or box-like patterns (Figures 2B and S4) [11,12,14,21]. In the case of diurnal and circadian time course data, the two most biologically relevant parameters are whether a transcript cycles and the timing or phase of its peak/trough of expression over the day. To capture both cycling and phase information in time course data, the patterns were modeled to 1-h increments over the day. For instance, the modeled spike pattern changes shape reflecting the anticipated peak in the 4-h resolution time course as it is shifted by 1-h increments (Figures 2C and S5).

Using 336 predefined patterns, we used HAYSTACK to interrogate the eleven time courses (Table 1, Figure S3). We established significance thresholds by permutation (Text S1). Two models, cosine and spike, were the most successful (highest correlation) at identifying cycling transcripts (Figure 2D). Traditionally, cycling activity has been detected using variations on spectral and sinusoidal analysis, which are based on fitting sine or cosine functions to data [27]. Comparison with one such method, COSOPT [11,28] revealed that on average HAYSTACK identified 86% of the cycling transcripts identified by COSOPT and 45% additional cycling transcripts than COSOPT (Table S1), mainly due to the inclusion of the spike pattern in the analysis. To test whether the enrichment of cycling transcripts identified by HAYSTACK was biologically relevant, we compared the amplitude (peak to average) of cycling transcripts to non-cycling transcripts. Despite the fact that HAYSTACK is amplitude-independent (being based on linear least squares regression), cycling transcripts had a higher amplitude change than non-cycling transcripts (Figure 2E).

**Figure 3:**
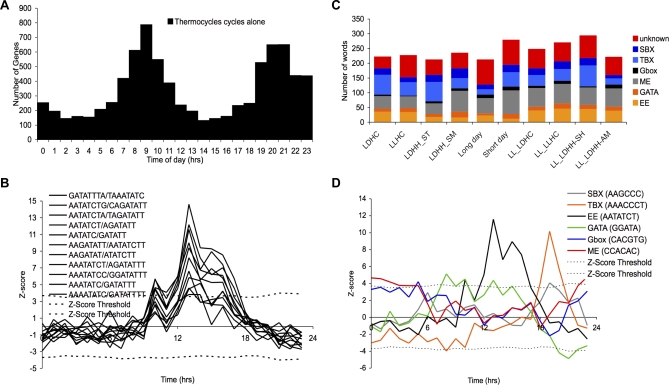
Diurnal and Circadian cis-Elements Identified over the Day (A) Frequency distribution of the number of cycling genes per phase bin under thermocycles alone. (B) z-Score profiles of overrepresented 3–8mer words composing the evening element (EE: AATATCT). z-Score threshold (dotted line) and z-score profiles (solid black lines) for words found in the EE. LLHC condition. (C) Number of words identified by condition. Unknown words (red), SBX, and TBX (shade of blue), Gbox/ME (shade of black), and EE/GATA (shade of orange). LLHC condition. (D) z-Score profiles of summarized words cover the entire day. SBX (grey), TBX (orange), EE (black), GATA (green), Gbox (blue), ME (red). LLHC condition.

The combination of the eleven diurnal and circadian conditions, with the 24 phases of the day, created 264 independent phase bins, each containing hundreds of co-regulated genes (Figure 3A). The list of genes in each phase bin served as the input for the enumerative promoter-searching tool ELEMENT, which identified overrepresented 3–8mer “words” in 500 bp of the upstream promoter regions [29]. The Web-based version of ELEMENT supports *Arabidopsis*, poplar, and rice and allows a user to choose various promoter lengths for analysis and to apply statistical filtering including adjusting the false discovery rate (FDR) [30,31].

ELEMENT was used to assign a significance z-score to each word for each bin. The z-scores were then plotted for each phase bin over the day creating a “z-score profile” for each time course, which in essence represents a serialization of the data (Figure 3B). To adjust for multiple testing, we applied a FDR to the one-tailed *p*-values corresponding to the observed z-scores. Doing so allowed us to establish a z-score threshold based on the equivalent corrected *p*-value. Since every 3–8mer word was tested for over-representation, similar z-score profiles often represent overlapping or nested words. For example, multiple words with similar z-score profiles overlap to define the previously characterized Evening Element (Figure 3B; EE; AATATCT) [11,16]. The z-score profile correctly predicts the EE phase of activity, and in addition provides novel information about flanking sequence due to the use of all 3–8mers.

The advantage of the z-score profiles is that they enable all 3–8mers to be evaluated based on specific hypotheses. To identify biologically relevant words across the z-score profile datasets, we applied our different patterns in HAYSTACK. We reasoned that biologically relevant words would have significant z-scores at more than one consecutive phase bin, and would be active at a particular phase of the day. By applying HAYSTACK to the z-score profiles of all 3–8mer words, we identified 2,185 unique 3–8 mers representing 200 to 300 words per condition. 75% of these words could be summarized into three groups of “elements,” which share both z-score profile and sequence similarity (Figure 3C, Table S2). Two groups, comprising 50% of all the words, could be summarized into four elements, EE, GATA element, G-box, and morning element (ME). All these elements were previously identified in light or circadian-associated studies [13,15–17]. The last group which constitutes 25% of the new words were summarized into two related elements, the telo-box (TBX: AAACCCT) [32] and a similar unknown element found in the larger maize sbe1 motif, named here the starch box (SBX: AAGCCC; Figure 3C and 3D) [33]. Words that comprise the TBX and SBX elements were found at the greatest frequency relative to other motifs across most conditions, only slightly greater than ME/Gbox (Figure 3C), while the EE had the highest z-scores across all conditions (Table S2). When the six elements making up the three groups are summarized, their predicted activity covers every phase of the day (Figure 3D). Therefore, using the network discovery pipeline we were able to predict cis-regulatory elements with confidence and to define specific aspects of their activity that were previously unknown.

### 89% of the *Arabidopsis* Transcriptome Cycles

**Figure 4:**
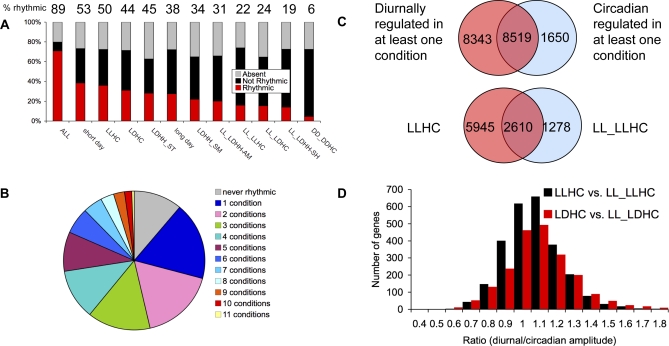
89% of the *Arabidopsis* Transcriptome Is Controlled by Thermocycles, Photocycles, or the Circadian Clock (A) Percentage of genes called rhythmic per condition. Grey bar represents the percentage of genes removed because they were called absent at more than nine time points over the twelve-time point time course. Red bars represent percentage of genes called rhythmic (r > 0.8; FDR ≤ 5.8%) using the model-based pattern-matching algorithm (HAYSTACK). Black bar represents the remaining genes that are not rhythmic (r < 0.8; FDR > 5.8%). % Rhythmic reflects the percentage of genes called rhythmic after exclusion of genes called absent. “ALL” represents genes that cycle in at least one of the eleven conditions. (B) Breakdown of percentage of genes that cycle per condition from “ALL” in (A). Genes that are never rhythmic represent 11% of the total genes that were called present in at least nine of twelve time points. The remaining 89% of genes were broken down by the number of conditions for which they were called cycling. For example, one condition means a gene only cycled under one of eleven conditions. (C) Circadian-regulated genes are a subset of diurnally regulated genes. Number of genes that overlap between genes called rhythmic under at least one diurnal condition (16,862) and genes called rhythmic under at least one circadian condition (10,169). Overlap between genes called rhythmic under LLHC compared to LL_LLHC. (D) Ratio of diurnal amplitude versus circadian amplitude. Amplitude was calculated as maximum unlogged gcRMA expression divided by the mean expression across the time course for a specific gene. Only genes that were rhythmic under both conditions were used in this analysis. LLHC versus LL_LLHC (black) and LDHC versus LL_LDHC (red).

Almost all (89%) of the reliably detected *Arabidopsis* transcripts on the Affymetrix ATH1 Genechip cycle under at least one of the 11 conditions tested (Figure 4A). Between 23% and 35% of gene models on the microarray were not statistically reproducible using the Affymetrix MAS5 present/absent call; these genes were not considered in the cycling analysis (Figure 4A, grey bars; Table S3). Within individual time courses, 34% to 53% of transcripts were diurnally regulated and between 6% and 31% were circadian regulated. The fewest cycling transcripts (6%) were detected under the continuous dark circadian condition, while the most cycling transcripts were detected under the diurnal conditions of short day photocycles and thermocycles alone (53% and 50%, respectively; Figure 4A). Most genes typically used as reference genes cycled under at least one condition (Table S4), leading us to create a new list of reference genes that have consistent expression but do not cycle across any of the 11 conditions tested (Table S5). 87 transcripts cycled under all 11 conditions (including continuous dark), which included most of the genetically defined circadian clock genes (Table S6 and S7), and some CONSTANS-like and CCA1/LHY-like genes that are thought to be circadian clock associated. While a diverse group of functions are represented in this gene list, the most highly overrepresented are transcription, energy, metabolism, and cell, organ, and tissue localization. Of particular interest are: *CDF3* and *PIF5*, which have recently been implicated in controlling growth in *Arabidopsis* [34], *SIGE*, an essential nuclear-encoded chloroplast targeted sigma factor [35], and the cyclin family protein (At1g27630), which provides a possible link between the circadian clock and the cell cycle.

Most genes cycle under a limited number of conditions, with 50% cycling under one to three conditions and 75% cycling under one to seven conditions (Figure 4B). On average, twice as many genes cycled under diurnal conditions as under circadian conditions (Figure 4A and 4C), consistent with the notion that circadian-regulated transcripts are only a subset of the transcripts that change abundance in response to thermocycles or photocycles. The set of transcripts that cycle under at least one circadian condition overlap with the set of transcripts that cycle under at least one diurnal condition (Figure 4C). However, there was less overlap between individual diurnal and circadian time courses (Figure 4C, Table S8). This result suggests that there is some specific circadian signaling that may be masked by the superimposed diurnal conditions. Consistent with diurnal cycles driving abundance of most transcripts, the amplitude of 50% of transcripts was higher under diurnal conditions compared to the associated circadian condition (Figure 4D). However, the amplitudes of 25% of the transcripts in the associated time course were higher under circadian conditions than under diurnal conditions, supporting the idea that sometimes superimposed diurnal regulation can reduce the level of circadian signal. In summary, we found that the majority of transcripts in *Arabidopsis* changed abundance over the day, either regulated by the circadian clock or directly controlled by daily environmental changes, consistent with the adaptive significance of appropriate synchrony with the environmental fluctuations.

### Transcripts Phased to All Times over the Day from Two Daily Set Points

**Figure 5:**
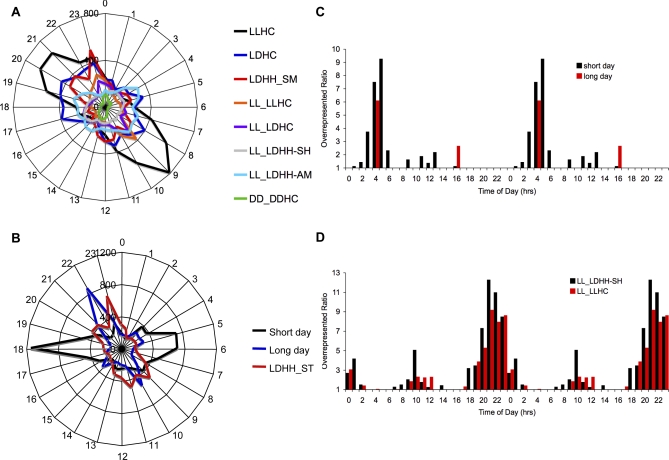
Transcripts Are Phased to Dawn and Dusk (A) Number of genes per phase under LLHC (black), LDHC (blue), LDHH_SM (grey), LL_LLHC (orange), LL_LDHC (purple), LL_LDHH-SH (grey), LL_LDHH-AM (light blue), and DD_DDHC (green). Radial plots with phase (h) on the circumference and number of genes on the radius. (B) Number of genes per phase under short day (black) and long day (blue) compared to LDHH_ST (red). (C) Phase overrepresentation plot comparing the phase under short day (black) and long day (red) of the 383 transcripts identified with the 2peak model under long days. Phase overrepresentation plots are generated by dividing the number of genes with a specific phase by the ratio of genes observed with that phase. [Number of genes in the list with phase X / (observed number of genes with phase X/total number of genes)]. (D) Phase overrepresentation plot comparing the phase under LL_LDHH-SH (black) and LL_LLHC (red) of the 383 transcripts identified with the 2peak model under long day.

To understand the global phase relationship between thermocycles, photocycles, and the circadian clock, we looked more closely at the global trends of phase across environmental conditions. Similar to other diurnal and circadian microarray reports in *Arabidopsis* [12,14], we found transcripts phased to every time over the entire day, and regardless of the condition, the majority of cycling transcripts preceded either dawn or dusk, separated by 12 h (Figure 5A and 5B). These results provide an additional confirmation concerning the phase relationship between external thermocycles or photocycles and transcript abundance. Thermocycles and photocycles set the global phase of the clock to the same reference time; in other words the cold to hot transition is analogous to dawn (the dark to light transition).

A previous study has shown that the circadian clock is involved in the control of dawn and dusk anticipation, which improves photosynthetic performance and increases fitness [2]. Our phase clustering results shows the extent of this control since the majority of the cycling genes peaks before dawn or dusk. This suggests that a large part of the gain in fitness conferred by the presence of the clock lies in the proper phasing of those early morning and evening genes. This model fits well with most of our phase data except under two conditions that both involve alternative photoperiods: long day (16 h light / 8 h dark) and short day (8 h light / 16 h dark) photocycles. Under long day photocycles, the large cluster of evening expressed genes preceded dusk by 6 h (compared to 2–3 h for other conditions), and under short day photocycles the large cluster of morning expressed genes preceded dawn by 6 h (Figure 5B). However, under both photoperiod conditions, the 12 h separation of the large clusters was maintained, as seen in all other conditions. This result is striking because it suggests two new aspects of the significance of phase. First, regardless of condition, the dawn/dusk co-regulated gene clusters maintain a 12 h phase difference. Second, photocycles play a dominant role in setting the phase of the dawn/dusk co-regulated gene clusters.

We noted transcripts that displayed two peaks over the day under both short and long day photocycles, and generally these transcripts were not called rhythmic by HAYSTACK. We reasoned that either these transcripts reflected overt biological rhythms shorter than 24 h, or circadian regulation that was split by photoperiod as predicted by the “morning and evening oscillator” model [36]. We constructed a 2-peak model, added it to HAYSTACK, and identified many transcripts displaying 2-peak phasing, with long day photocycles having the largest number (Table S9; Text S1). We found 383 transcripts that displayed 2 peaks under long day photocycles and only 67 of them had originally been called rhythmic by HAYSTACK (Table S9, Figure S6). To test if the transcripts detected in the long day time course were circadian regulated in other conditions and at which phase they were expressed, we generated phase over-representation plots that normalized the number of transcripts in a list with a specific phase, by comparing them to the expected number of transcripts at that phase (Text S1). Of the 383 transcripts that we found with the 2-peak model under long day photocycles, the majority had mid-day specific expression under either short or long day photocycles as called by HAYSTACK without the 2-peak model (Figure 5C). In addition, these transcripts were controlled by the circadian clock and specifically phased to dawn and dusk (Figure 5D). These results suggest that the long photoperiod 2-peak transcripts are in fact circadian regulated, and adds support to the notion that the *Arabidopsis* circadian network may be composed of photoperiod sensitive morning and evening oscillators [37].

### Gene-Specific Phase Shifts

**Figure 6:**
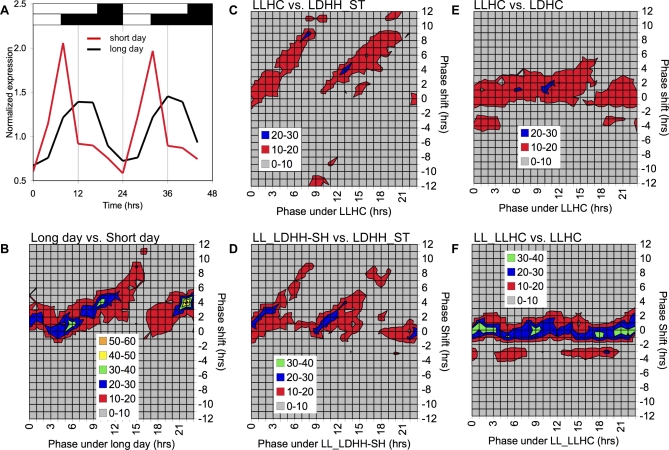
Thermocycles and Photocycles Have Distinct Phase Relationships (A) Long day photocycles (16 h light/8 h dark) phase delay genes compared to short day photocycles (8 h light/16 h dark). Expression pattern is the average of 23 genes displaying a 6-h phase delay between long day (black, phase 13 h), and short day (red, phase 7 h). (B) Long day photocycles globally phase delay genes as compared to short day photocycles. Phase shift topology graph plots percent of genes phase shifted per phase bin (*y*-axis) by the reference condition phase (*x*-axis). Only genes that are rhythmic between both conditions are used in this analysis. Percent of genes was calculated as the number of genes with a given phase shift per phase divided by the total number of genes with that phase. A positive phase shift reflects a later phase than the reference condition, and a negative phase shift reflects an earlier phase than the reference condition. Long day photocycle is the reference condition and consistent with (A), long day photocycles delay the phase (positive phase shift). (C) Phase-shift topology between LLHC and LDHH_ST, where LLHC is the reference phase. (D) Phase-shift topology between LL_LLHH-SH and LDHH_ST, where LL_LDHH-SH is the reference phase. (E) Phase-shift topology between LLHC and LDHC, where LLHC is the reference phase. (F) Phase-shift topology between LL_LLHC and LLHC, where LL_LLHC is the reference phase.

No study to date has addressed the problem of how individual phase relationships of thousands of genes are affected by photocycles, thermocycles, and the circadian clock. To address this question, we constructed “phase topology maps” to compare the phase of individual transcripts between two environmental conditions (Figure 6). *CHLOROPHYLL A/B BINDING* protein (*CAB*) gene expression is phased to the middle of the photoperiod irrespective of day length, so a 4-h phase shift relative to dawn is apparent when the photoperiod is extended by 8 h (Figure 6A) [38]. Consistent with *CAB* expression, a higher percentage of transcripts were shifted to a later phase under long day photocycles (Figure 6B). For example, transcripts peaking at 10 h and 22 h after dawn under long day photocycles were shifted by 4 h, since they peaked at 6 h and 18 h after dawn under short day photocycles, respectively. One might predict from *CAB* gene expression that the entire transcriptome would shift by 4 h; however, this was not the case. Not all transcripts shifted phase between short day and long day photocycles. Transcripts phased to 10 h and 22 h after dawn under long day photocycles displayed the largest shift under short day photocycles. The number of hours the phase of a transcript was shifted correlated with the distance it was phased from either midday or midnight, resulting in a “skewed” linear phase shift topology.

A similar but more dramatic skewed linear phase-shift topology was observed between thermocycles alone (LLHC) or circadian conditions (LL_LDHH-SH) compared to photocycles alone (LDHH_ST; Figure 6C and 6D). We reasoned that this dramatic phase shift might reflect the release into continuous light, either on the initial transfer (LLHC) or after entrainment (LDHH_ST), mimicking a phase delay associated with a day length extension. Consistent with this, LDHH_ST and long day photocycles did not have a skewed linear phase-shift topology. Consequently, some genes, but not all, are set by the last dark to light transition. The highest percentage of transcripts that did not shift between LLHC and LDHH_ST had phases at either dawn or dusk. A uniform pattern emerged, similar to short and long photocycles, with the magnitude of the shift equal to the distance from dawn or dusk. In contrast, only 10% to 20% of the transcripts phased to 12 h to 16 h after dawn displayed the skewed linear phase-shift topology between LLHC and LDHC (Figure 6E), suggesting that when photocycles and thermocycles are superimposed, thermocycles dominate to set the phase of the midnight expressed transcripts. Thermocycles phased most transcripts to the same time of day as circadian conditions (Figure 6F), whereas photocycles dramatically shifted specific circadian regulated transcripts from the dawn and dusk set points (Figure 6D).

The phase topology results revealed multiple important aspects of phase-specific expression. First, thermocycles and the circadian clock phased transcripts to the same phase of the day, consistent with thermocycles acting through the circadian clock to set phase [8–10]. In contrast, photocycles antagonize the phase of the clock and shift transcripts to a new phase of the day; this photocycle-dependent shift is the same seen in growth rhythms [34,39–41]. Second, there are two reference points during the day from which all genes are shifted as predicted by the morning and evening oscillator model [36]. This finding demonstrates the novelty and the importance of comparing environment-specific phase changes across individual transcripts with phase topology graphs. Finally, while the general trend is that thermocycles and photocycles phase transcripts to anticipate dawn and dusk, a closer look at individual transcripts revealed that thermocycles and photocycles play distinct roles in establishing phase.

### Protein Synthesis Controlled by Thermocycles

**Figure 7:**
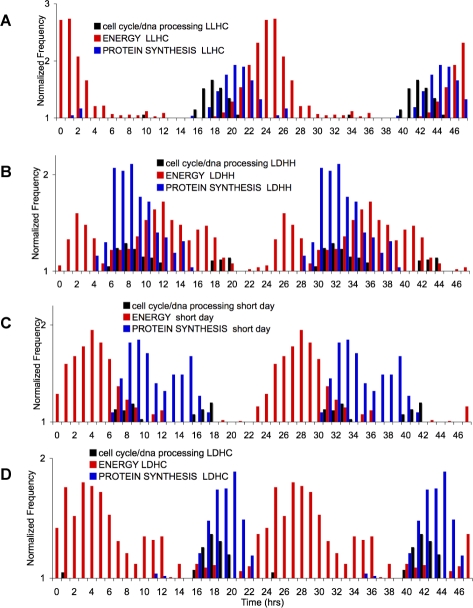
Thermocycles Phase Protein Synthesis to Midnight (A–D) Protein synthesis genes are overrepresented at distinct times of day under thermocycles and photocycles. Three consecutive phases were merged and used as the input genes list per phase. The data are double plotted (one day of data displayed as two days) for visualization purposes. Cell cycle/DNA processing (black), protein synthesis (red), and energy (blue) genes plotted as normalized frequency. Normalized Frequency is calculated as follows: Number_in_Classinput_set/Number_Classifiedinput_set)/(Number_in_Classreference_set (ATH1)/ Number_Classifiedreference_set). Gene Ontology overrepresentation maps were made using the Classification SuperViewer Tool at Botany Array Resource (http://bbc.botany.utoronto.ca/). (A) LLHC; (B) LDHH-ST; (C) short day; (D) LDHC.

To determine at what time-of-day–specific biological processes occur and how different environmental conditions affect their time of day activity, we queried every phase bin to see whether some gene ontology categories were overrepresented. Using the Classification SuperViewer Tool with Bootstrap at the Bio-Array Resource for *Arabidopsis* Functional Genomics (http://bbc.botany.utoronto.ca/), we calculated the normalized frequency for each gene ontology category for each phase bin. Following this procedure, we double plotted the normalized frequency and searched for time-of-day–specific patterns in the gene ontology categories. We found that the gene ontology categories of “cell cycle/dna processing”, “energy”, and “protein synthesis” were phased between midnight and dawn under thermocycles in constant light (Figure 7A). Under conditions with no thermocycles (any condition with a photocycle), these gene ontology categories were phased to midday (Figure 7B and 7C). Even under photocycles and thermocycles together, these gene ontology categories were phased to the same time of day as thermocycles alone (Figure 7D), supporting the idea that thermocycles dominate over photocycles to regulate the genes in these categories. In contrast, photocycles preferentially drive genes from the “energy” category; which are phased before dawn under thermocycles alone, and after dawn when there is a photocycle (Figure 7A and 7B). It should be noted that it is only when thermocycles and photocycles are superimposed that energy is clearly partitioned from cell cycle and protein synthesis. These results suggest that thermocycles synchronize processes such as cell cycle and protein synthesis so that they precede the daily dawn specific growth cycle [34,39–41]. In fact, when plants are treated with opposing thermocycles and photocycles (cold days and warm nights), growth is reduced by 75%, despite experiencing the same amount of temperature and light [42,43]. This supports the idea that the timing of thermocycles and photocycles plays an essential role in growth regulation.

### Time-of-Day–Specific cis-Regulatory Modules

**Figure 8:**
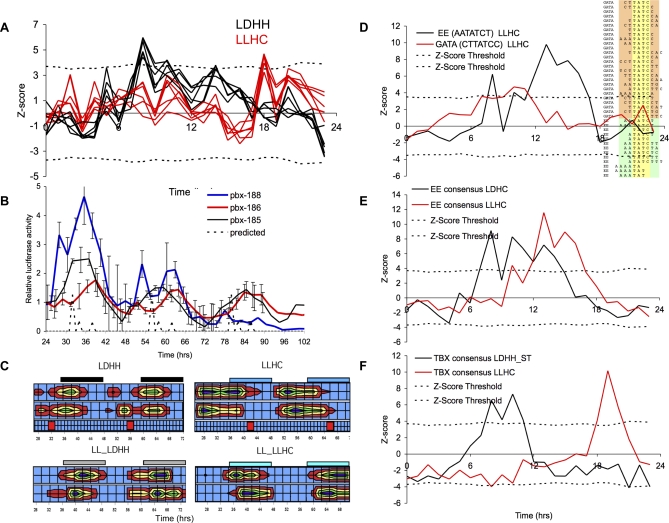
The PBX/TBX/SBX cis-Regulatory Module Controls Condition Specific Diurnal and Circadian Transcription (A) z-Score profiles of words that make up the PBX (ATGGGCC) under LDHH (black) and LLHC (red). (B) 3xPBX::LUC cycles under LDHH. Four to six seedlings from several independent T2 lines were analyzed and averaged. Results from three independent experiments are shown (Experiment number 185 *n* = 10, 186 *n* = 4, and 188 *n* = 3). (C) PBX cycles under all diurnal and circadian conditions tested. Heat map displaying the phase of the 3xPBX::LUC lines from two independent experiments under four different experimental conditions, LDHH (black bar, dark), LLHC (blue bar, cold), LL_LDHH (grey bar, subjective night), and LL_LLHC (light blue bar, subjective night). The predicted phase for LDHH and LLHC is displayed below heat maps (red square). Heat map from high relative expression to low (purple, green, yellow, red, and blue). (D) EE (black) and GATA (red) z-score profiles have distinct phases of overrepresentation, and share the GATA core but differ at flanking sequence. (E) z-Score profile of the consensus EE shifts phase between LDHC (black) and LLHC (red). (F) z-Score profile of the consensus TBX shifts phase between LDHH_ST (black) and LLHC (red).

To biologically validate our predictions of cis-regulatory elements from the network discovery pipeline, we tested if a multimer of the unknown element, ATGGGCC, was sufficient to confer diurnal and circadian activity to a luciferase reporter. The ATGGGCC z-score profile corresponded to the phase of protein synthesis transcript abundance under thermocycles and displays sequence, as well as z-score profile similarity, to the TBX and SBX (Figure 8A and 8F). A scan of *Arabidopsis* promoters (500 bp) revealed 1,732 occurrences of ATGGGCC in 1,541 genes, which were enriched for protein synthesis gene ontology annotations. Based on these findings, the ATGGGCC was named the protein box (PBX). To validate the PBX, we designed a fusion construct with the PBX in triplicate preceding the minimal nos promoter driving luciferase (3xPBX::LUC). Multiple independent T2 lines carrying 3xPBX::LUC conferred diurnal and circadian regulation to luciferase activity in vivo under every condition tested (Figures 8B and 8C and S8). The luciferase activity of the 3xPBX::LUC displayed condition-specific activity consistent with our phase predictions (Figure 8C). Thus, we have identified a new circadian and diurnal response element conferring midnight expression. This element may be considered the plant counterpart to the Rev-ErbA/ROR element (RRE) in mammals [44] due to its midnight-specific activity.

The TBX, SBX, and PBX are overrepresented at midnight, a time of day lacking predicted light or circadian elements in *Arabidopsis*, and, in combination with the EE, GATA, ME, G-box elements, these element are predicted to cover every phase of the day (Figure 3D). However, we noted that despite their distinct z-score profiles across conditions, the EE and GATA, and the ME and Gbox shared core sequence while differing at flanking sequence. The GATA box and the EE share the GATA core (TATC), and differ at flanking sequences (CTtatcC versus AAtatcT), while having distinct overrepresentation at 10 h and 13 h after dawn, respectively (Figures 3D and 8D). We also noted a similar situation with the ME and the Gbox (NccacACN versus GccacGTG). The ME and G-box share the CCAC core, yet differ at flanking sequence and were overrepresented before dawn and after dawn, respectively (Figures 3D and S7). The EE/GATA and ME/Gbox can be thought of as two “phase modules,” where core sequence specifies time of day and flanking sequence refines the exact phase in a transcription factor or environmental specific fashion. Consistent with this idea, we noted that the EE/GATA displays a condition-dependent phase of overrepresentation between photocycles and thermocycles (Figure 8E). In contrast to the dawn/dusk modules, the elements of the midnight-specific module (PBX/TBX/SBX) differ at core sequence and share flanking sequence (aaaCCC/aagCCC/tggCCC). The midnight module displays antiphasic overrepresentation between photocycles and thermocycles (Figure 8A and 8F). Together, the three phase modules cover the entire day, displaying striking similarity to the core circadian network described in mammals [45].

### Time-of-Day cis-Modules Are Conserved across Species

**Figure 9:**
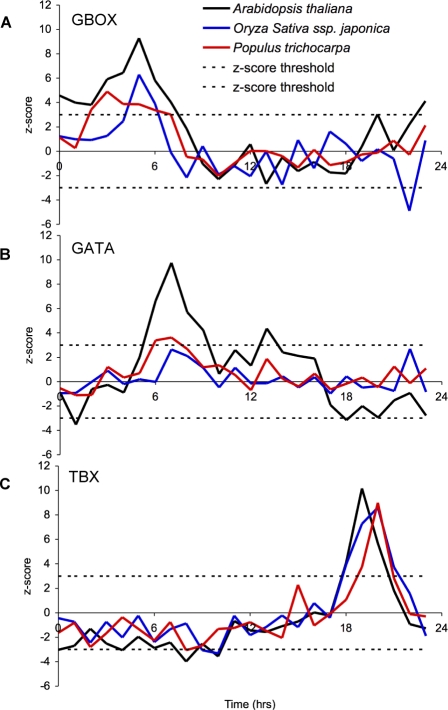
The Three cis-Regulatory Modules Are Conserved across Species z-Score profiles of cis-regulatory modules in Arabidopsis thaliana (black), *Oryza sativa ssp. japonica* (rice, blue), and Populus trichocarpa (poplar, red). z-Score threshold (dotted line). (A) z-Score profile of the Gbox (CACGTG). (B) z-Score profile of the GATA (GATA). (C) z-Score profile of the TBX (AAACCCT).

The conservation of network structure between *Arabidopsis* and mammals led us to speculate that we could extend the network discovery pipeline to distantly related plant species. We reasoned that if specific time-of-day transcriptional networks were conserved between species, then the cis-regulatory elements may also be conserved. To test this, we used all-versus-all reciprocal BLASTP analysis to identify putative *Arabidopsis*-poplar and *Arabidopsis*-rice orthologs using the “mutual-best-blast-hit” criteria [46]. To assess the conservation of promoters between orthologs, we used BLAST (bl2seq) to directly compare promoters (500 bp upstream of the ATG) of orthologous pairs. We found that only short DNA sequences (≤8 mers) were shared between orthologous promoters, suggesting that cis-elements could be conserved (Figure S9). To determine if we could detect conserved cis-elements, we assigned the phase of transcript abundance from *Arabidopsis* to its corresponding ortholog in rice and poplar. In other words, rice and poplar orthologs were organized into phase bins based on cycling in *Arabidopsis*. We then used this phase information to seed ELEMENT to find cis-regulatory motifs within rice and poplar promoters (500 bp upstream of the ATG). Using only the orthologous phase information as a predictor in rice and poplar, we found that the timing and overrepresentation of the three cis-regulatory modules were similar across these three species (Figure 9). These data suggest that both transcriptional networks and time-of-day specific biological processes are well conserved across distantly related plant species.

## Discussion

In this study, we present a framework for de novo prediction of system dynamics and transcriptional circuits in diurnal and circadian biological networks. Our analysis elucidated transcriptional circuitry that regulates phase-specific modules mediating the interaction between external thermocycles, photocycles, and the internal circadian clock. We found that most *Arabidopsis* transcripts cycle under the diverse diurnal and circadian conditions tested. We confirmed known cis-acting elements and their specific time of activity while expanding both their sequence and phase definitions. We identified and validated a new mid-night cis-element, and found that the predicted activity of these time-of-day–specific cis-elements is conserved across distantly related species.

We identified transcriptional circuitry mediated by at least three phase regulatory modules, ME/G-box, EE/GATA, and PBX/TBX/SBX (Figure S10), which parallel the three mammalian cis elements, Ebox, Dbox, and RRE [45]. The mammalian circadian transcriptional network is controlled by two design principles: “repression precedes activation” and “repression is antiphasic to activation” [45]. The “repression precedes activation” principle predicted *in silico* that close temporal binding (1 h to 3 h) of activators and repressors to cis-elements results in either moderate phase delays or phase advances in expression. We predicted moderate phase shifts (3 h to 6 h) from dawn and dusk based on the flanking sequence around the ME/Gbox and EE/GATA modules, respectively. Our findings extend this principle by suggesting that evolution of specific flanking sequences provides the promoter context necessary for the phase modularity generated by close temporal binding of activators and repressors.

The second principle, “repression is antiphasic to activation” predicts that activators and repressors bind cis elements in antiphase (12 h apart), resulting in greater amplitude of transcriptional activity. We predicted that the third module, PBX/TBX/SBX, was active at midnight under any condition with thermocycles (and circadian conditions), and 12 h early (antiphase) under any condition without thermocycles (photocycles alone). Since plants rarely experience photocycles without thermocycles in nature, the antiphase activity of this module under the condition of photocycles alone suggests that the nature of this module is consistent with the large (12 h) phase shifts predicted by the second principle. AtPurα (At2g32080), whose homologs were implicated in cell cycle timing in multiple model systems, binds the TBX *in vitro* [47], and the phase of its transcript is antiphase (12-hr shift) between photocycles and thermocycles alone as well. The TBX was originally identified in interstitial telomere repeats with overrepresentation in *eEF1A* genes in *Arabidopsis* [32], and in combination with other cis elements, it is involved in cell cycle regulation and sugar signaling [48–50]. The TBX/SBX/PBX module provides a mechanistic link between the circadian clock and cell cycle progression where DNA replication is phased to midnight limiting the coincidence with harmful UV irradiation [51,52]. The greater amplitude of transcriptional activity predicted by the second principle is consistent with the TBX/SBX/PBX module ensuring temporal synchrony of the cell cycle and the circadian clock, processes essential for enhanced fitness and adaptation.

A three-loop network has been proposed for the *Arabidopsis* circadian clock [37,53] (Figure S10). Similar to the *Drosophila* and mammalian network architecture, a three-loop model suggests that the *Arabidopsis* network may be composed of both morning and evening specific oscillators that are coupled, allowing for photoperiod sensitivity [36,37]. In this study, the phase topology maps and 2-peak analysis support morning and evening oscillatory mechanisms (reference points) from which all genes are phased. In addition, we show that environmental conditions selectively shift the phase of these two oscillatory mechanisms consistent with them being separable as predicted by analysis of *Arabidopsis* clock mutant phenotypes. The *Arabidopsis* circadian clock has a temperature sensitive oscillator that can be distinctly phased from a light sensitive oscillator [7]. Similarly, we found that thermocycles set the phase of the circadian clock, independent of photocycles, creating an internal phase relationship between specific sets of transcripts. The circadian clock governs the coincidence of internal and external cycles controlling important processes such as reproductive timing (flowering) in *Arabidopsis* [54]. Under conditions in which *Arabidopsis* does not flower, short day photocycles, we found that the morning and evening oscillatory mechanisms establish a unique phase relationship with all other conditions tested. This provides another example of the importance of “external coincidence” in biological timing and the regulation of plant growth and development.

About 90% of the *Arabidopsis* transcriptome cycles under at least one condition of thermocycles, photocycles, or the circadian clock. In retrospect, this result is not surprising since plants must accurately anticipate daily changes in their environment [1–3]. This large number of cycling transcripts may represent multiple levels of regulation and the cycling of rate-limiting protein complexes. While it is possible that the large number of cycling transcripts reflects experimental artifacts, recent re-analysis of old data suggest that most mammalian transcripts cycle [55]. Global transcriptional regulation by the circadian clock in *Arabidopsis* is reminiscent of the cyanobacterial clock where all transcripts are under the regulation of the circadian clock [56]. Despite global circadian regulation, a two-component regulatory system in cyanobacteria is necessary to mediate the phase-specific expression required for optimized growth under photocycles [57]. Indeed, global diurnal and circadian changes in transcript abundance may reflect underlying rhythms in chromatin structure or modifications as seen in the mammalian and cyanobacterial systems [58–62]. This regulation may be centered on key cis-elements since the Ebox, and its transcriptional activators BMAL1 and CLOCK, are required for chromatin modifications and circadian transcription in the mammalian system [63].

The maximal phase of stem growth in *Arabidopsis* occurs at dawn under photocycles alone [34,40,41], while growth is shifted to dusk under circadian conditions [39] or thermocycles alone (TPM and JC, unpublished results). In fact, when plants are treated with thermocycles and photocycles in antiphase (cold days and warm nights), growth is severely inhibited [42,43], consistent with the specific roles these conditions play in synchronizing growth-related pathways. We found that under any condition with photocycles, energy and protein synthesis genes were overrepresented after the maximal growth phase at dawn, suggesting that photocycles act to partition growth and photosynthesis. However, thermocycles play a distinct and specific role, phasing protein synthesis genes to midnight, preceding the growth phase under this condition, regardless of photocycles. In nature, plants rarely experience photocycles without a corresponding thermocycle, and some developmental processes such as seed germination occur under thermocycles alone [64]. We demonstrated that thermocycles alone controlled more than 50% of the *Arabidopsis* transcripts, 28% of which cycle only under thermocycles alone, supporting the important role that thermocycles play in setting the internal phase of the plant. Similarly, thermocycles control a large number of transcripts in *Drosophila* [25], corroborating the importance of thermocycles across species. It is tempting to predict that thermocycles act to set the phase of the circadian clock, while external photocycles are superimposed, leading to accurate seasonal estimation and appropriate growth/developmental patterns [65].

While the pipeline allowed us to uncover new aspects of the time-of-day–specific transcriptional network, the scale of the dataset provides new insights into how photocycles and thermocycles interact with the circadian clock to govern essential biological functions. Microarray data in *Arabidopsis* and Drosophila have revealed that the majority of transcripts anticipate dawn and dusk [12,14,25]. However, our photoperiod data where the 12-h phase difference is maintained over anticipating environmental changes, suggests phase is a fundamental parameter of the oscillator. Underlying the core 12-h phase differences are critical phase nodes, such as the ME/Gbox and EE/GATA. The phase modules are a fundamental aspect of the core clock, always maintaining their relationship despite external conditions. Furthermore, thermocycles act independently of photocycles on processes such as cell cycle, protein synthesis, and DNA replication, possibly through elements such as the PBX/TBX/SBX. Together, thermocycles and photocycles, which are almost always present together in nature, interact to partition biological activities to the correct times over the day. Thus, our pipeline, a conceptual platform that couples new approaches with well-developed methodologies, uncovers novel network topologies and their underlying components.

## Material and Methods

### Plant material, growth conditions, and time courses.

The time courses are summarized in Table 1 and Figure S3. Arabidopsis thaliana seedlings, reference accessions Columbia (Col-0), or Landsberg *Erecta* (L*er*) were sterilized, plated on ms agar media plus and minus sucrose, stratified for four days at 4 °C, and released into the specified condition. Temperature and light cycles were monitored every 5 min and recorded using HOBO data recorders (Onset). LDHH_ST, LDHH_SM, and LL_LDHH-AM time courses were previously described [12,14,21]. The two replicates of the LDHH_ST and LDHH_SM time courses were averaged and double plotted to be parallel to the other time courses.

### RNA preparation, cRNA synthesis, and microarray hybridization.

All microarray techniques were per manufacturer-supplied protocols. RNAs were extracted from frozen tissues, and labeled probes were prepared and hybridized to Affymetrix *Arabidopsis* ATH1 Genechip per Affymetrix protocols (Affymetrix).

### Array quality control and normalization.

We checked array quality using standard tools implemented in the Bioconductor packages simpleaffy and affyPLM. All 132 microarrays were normalized together using gcRMA. Present/absent calls were made using the Affymetrix MAS5 program (Affymetrix).

### HAYSTACK: Model-based pattern-matching algorithm.

HAYSTACK, a model-based pattern-matching algorithm, compares a collection of diurnal/circadian models against microarray time-course data to identify cycling genes (Figures S4 and S5). HAYSTACK has been implemented in perl, and uses least-square linear regression for each gene against all model cycling patterns with 24 possible phases. A series of statistical tests were used to identify the best-fit model, phase-of-expression, and to estimate a *p*-value and false-discovery rate (FDR) [30,31] for each gene. We selected cycling genes using a correlation cutoff of 0.8, which corresponds to a maximum FDR of 3.1% to 5.8% in different datasets. HAYSTACK can be accessed online at http://haystack.cgrb.oregonstate.edu.

### ELEMENT: Enumerative promoter searching.

We established a cis-regulatory element analysis pipeline to identify the putative promoter sequences upstream of these genes (Figure 3). This platform comprises databases of putative *Arabidopsis,* rice, and poplar regulatory DNAs, word statistics for all 3–8mer DNA words occurring in these promoter sequences, software (http://element.cgrb.oregonstate.edu) implemented in perl to analyze promoters and apply statistical screening criteria, and a series of accessory scripts to summarize the results of these analyses.

### Synthetic luciferase promoter fusions, *Arabidopsis* transformation, and luciferase imaging.

The 3xPBX::LUC and UNDER1:LUC constructs were made by ligating two long oligos containing the PBX or UNDER1 into a vector containing the −101/+4 fragment of the NOS minimal promoter and modified firefly luciferase (luc+) as reported previously [15] (Text S1). Analysis of several T1 plants transformed with the empty plasmids revealed that there was no emitted bioluminescence, suggesting that the plasmid backbone didn't contain a DNA motif that could drive the luciferase reporter (unpublished data). Plasmids were transformed into the Col-0 accession using the floral dip method. Except where indicated, seedlings were grown on MS medium (Gibco BRL) with 0.8% agar and 3% sucrose. Seedlings of the T_1_ generation were selected on kanamycin and transferred to soil for propagation. T_2_ seedlings were grown without selection before imaging. Wild-type seedlings were identified after image collection and removed from the analysis. During the initial week of growth, seedlings were grown under LDHH conditions. Two or three days prior to imaging, seedlings were transferred to the proper entrainment condition (LDHC or LDHH or LLHC) on smaller plates without sucrose. Images of seedlings were collected over the course of five days using a cooled CCD camera for 25 min every 2.5 h using the Wasabi software (Hamamatsu Photonics) in the slice photoncounting mode. The images were quantified using the MetaMorph software (Universal Imaging) and graphed using Microsoft Excel (Microsoft). For each independent T2 line, four to six seedlings were analyzed per experiment. To allow comparison with other T2 lines, each value was divided by the median value of the whole time course. The relative bioluminescence values were averaged for the progeny of each T2 line. Three to ten independent T2 lines were used for each experiment. For data display, we generated an average of the average, which combined the values from the four to six seedlings from the three to ten T2 lines analyzed.

## Supporting Information

Figure S1 Daily Interactions of Temperature and Light in Nature by Month Hourly temperature (°C, dotted line) and light (solar radiation, watts/m2, filled line) data were obtained for Princeton, Kentucky, United States, from the Soil Climate Analysis Network (http://www.wcc.nrcs.usda.gov/scan/); SCAN site number: 2005; latitude: 37° 06′ N; longitude: 87° 50′ W; elevation: 615 feet. Hourly data from April over three consecutive years were reformatted and averaged by hour over the month, and plotted over the 24-h day. Data are averaged by month and day over three years. Daily relationship between temperature and light during an average day in April (A), October (B), July (C), and January (D).
(108 KB TIF)

Figure S2 Daily Interactions of Temperature and Light in Nature by Latitude Hourly temperature (°C, dotted line) and solar radiation (watts/m2, filled line) data were obtained from the Soil Climate Analysis Network (http://www.wcc.nrcs.usda.gov/scan/). The data were analyzed as described in Figure S1.
(A) Daily relationship between temperature and light during an average day in April. SCAN site number: 2010; Newton, Newton County, Mississippi, United States; latitude: 32° 20′ N; longitude: 89° 05′W; elevation: 300 feet. Data average from April of 1999.
(B) Daily relationship between temperature and light during an average day in April. SCAN site number: 2005; Princeton, Kentucky, United States; latitude: 37° 06′ N; longitude: 87° 50′ W; elevation: 615 feet. Data average from April of 1999.
(C) Daily relationship between temperature and light during an average day in April. SCAN site number: 2043; Mascoma River, Grafton County, New Hampshire, United States; latitude: 43° 47′ N, longitude: 72° 02′ W, elevation: 14 feet. Data average from April of 2000.
(89 KB TIF)

Figure S3 Sampling Strategy for Diurnal and Circadian Time Courses Light: white boxes represent the “lights on” conditions and black boxes represent “lights off”. Temperature: blue boxes represent low temperatures (12 °C), and white boxes represent high temperatures (22 °C). Note, two replicates were sampled over one day for both LDHH_ST and LDHH_SM, and sampling began in the evening for the later time course. Also, samples were collected on days two and three starting at 26 h (CT2) after subjective dawn, as compared to all other time courses that that start at CT0. Grey boxes represent subjective night or cold during circadian time courses.
(181 KB TIF)

Figure S4 Models Used To Identify Diurnal and Circadian Regulated Transcripts (A) Rigid, (B) Spike, (C) Cosine, (D) Box2, (E) Box1, and (F) Asymetrix rigid model (asyrigid).
(76 KB TIF)

Figure S5 Models Shifted by 1-h Increments To Quantify Time of Transcript Abundance All models were shifted in 1-h increments causing a change in the model form and shape over the day to account changing waveforms caused by our 4-h sampling strategy. The models are presented in batches covering 4-h time intervals for presentation purposes.
(A) The spike model shifted to ZT00, ZT01, ZT02, and ZT03.
(B) The spike model shifted to ZT04, ZT05, ZT06, and ZT07.
(C) The spike model shifted to ZT08, ZT09, ZT10, and ZT11.
(D) The spike model shifted to ZT12, ZT13, ZT14, and ZT15.
(E) The spike model shifted to ZT16, ZT17, ZT18, and ZT19.
(F) The spike model shifted to ZT20, ZT21, ZT22 and ZT23.
(162 KB TIF)

Figure S6 2Peak Model Identifies Transcripts Under Long Days (A) Number of transcripts called by the 2peak model across conditions.
(B) The expression pattern of PHOT1, which was called by the 2peak model under long day photocycles, under short day (black solid), long day (black dash), and LDHH_ST (grey).
(71 KB TIF)

Figure S7 Flanking Sequence Distinguishes the G-Box from the Morning Element All words identified in the cis-regulatory element analysis pipeline that overlap with the morning element (ME) were aligned. Two classes of elements emerged based on flanking sequence that share a CCAC core (yellow shading). The distinguishing sequence is highlighted for both ME (purple) or the Gbox (blue), and were summarized ccacAC and ccacGTG, respectively. Graphing of the z-score profiles of ccacAC (blue) and cacGTG (red) predict that they have distinct temporal activity. ccacAC displays maximum overrepresentation 23 h after dawn, and cacGTG displays maximum overrepresentation 4 h after dawn.
(261 KB TIF)

Figure S8 Multiple PBX::LUC Show Rhythmic Behavior Under LDHH (A) Ten independent T2 lines out of ten display rhythmic behavior under LDHH (experiment TM185 for which average trace is displayed on Figure 8B).
(B) Four independent T2 lines out of four display rhythmic behavior under LDHH (experiment TM186 for which average trace is displayed on Figure 8B).
(C) Z-score profile of the negative control element UNDER1 (GGACGTAC) under LLHC. The under1 element doesn't exhibit phase overrepresentation under any conditions tested.
(D) Bioluminescence of five independent T1 lines of UNDER1::LUC under LLHC.
(E) *p*-Values derived from all the T2 progeny for the PBX::LUC analyzed in three independent experiments. Included is the *p*-value for the positive (EE-CCR2::LUC) and negative (UNDER1::LUC) controls.
(175 KB TIF)

Figure S9 Only Short DNA Segments Conserved between Promoter Regions of *Arabidopsis*, Rice, and Poplar Promoter regions (500 bp) were aligned using BLASTN (bl2seq) between *Arabidopsis* and poplar (black) or *Arabidopsis* and rice (red).
(157 KB TIF)

Figure S10 Circadian Transcriptional Network Model The central feedback loops are an approximation of the circadian clock network in *Arabidopsis*. Timing is entrained by input signals such as photocycles and thermocycles. Rhythmic transcription factors act upon three important regulatory motif groups (the ME/G-box, GATA/EE, and the PBX/SBX/TBX) via transcription factors. Photosynthesis and protein synthesis/cell cycle are timed to occur in the morning and evening, respectively. Diurnal regulated growth occurs in the morning, while specifically clock regulated growth occurs in the evening.
(274 KB TIF)

Table S1 Comparison of Genes Called Rhythmic between HAYSTACK and COSOPT (172 KB TIF)

Table S2 All Significant Words Identified in the Promoter Pipeline (1.7 MB XLS)

Table S3 Genes Call Absent by Affymetrix MAS5 Algorithm (245 KB TIF)

Table S4 Phase of Commonly Used Reference Genes (163 KB TIF)

Table S5 Possible Reference Genes with Good Expression That Do Not Cycle (28 KB XLS)

Table S6 Phase of Known Circadian Clock Genes in This Study (246 KB TIF)

Table S7 Genes That Cycle Under All 11 Diurnal and Circadian Conditions Tested (49 KB XLS)

Table S8 Percent Overlap between Diurnal and Circadian Regulated Genes Percentage of genes captured by the condition at the top on the lower diagonal, and the percentage of genes captured by the condition on the left on the top diagonal. For instance, LLHC captures 70% of the genes in LL_LLHC (lower diagonal), and LL_LLHC captures 33% of the genes in LLHC (upper diagonal).
(220 KB TIF)

Table S9 Transcripts Called with the 2peak Model Across All 11 Conditions (204 KB XLS)

Text S1 The Interaction between Thermocycles and Photocycles in Nature (133 KB DOC)

### Accession Numbers

All data has been deposited at ArrayExpress under accession number E-MEXP-1304. These data are also available online at http://diurnal.cgrb.oregonstate.edu.
