# Supplementary material for: Network Discovery Pipeline Elucidates Conserved Time-of-Day–Specific cis-Regulatory Modules
Source: PLoS Genet. 2008 Feb 1;4(2):e14. doi: 10.1371/journal.pgen.0040014 (PMC2222925; doi:10.1371/journal.pgen.0040014)
Supplement: Supplementary file 1 [file pgen.0040014.sd001.doc]

**Supporting Information**

**The interaction between thermocycles and photocycles in nature**

In the external environment, there is a dynamic daily and seasonal relationship between light (photocycles) and temperature (thermocycles) depending on latitude (Figure S1, S2). For example, at 37°N latitude, where *Arabidopsis* populations commonly grow, the average April air temperature increases with solar radiation, reaches a maximum a few hours after the solar maximum, and then decreases much more slowly with the thermal minimum during the night (Figure S1). However the daily relationship between photocycles and thermocycles changes based on season (Figure S1) and latitude (Figure S2). All raw environmental data were downloaded from the USDA SCAN web site and formatted with an excel macro. Raw data are available in one-hour increments for multiple years. Daily solar and temperature information reflects the average of each day over the month. The average monthly value was then averaged by month over three separate years.

**HAYSTACK: model based pattern-matching algorithm**

Several algorithms have been utilized to identify genes that exhibit diurnal and circadian gene expression in microarray time-courses in *Arabidopsis*, Drosophila, and mammalian systems [1,2]. In general, microarray time course data lack both dynamic range and temporal resolution, resulting in expression patterns that appear driven, not smooth. We found that diurnal microarray data conformed to a set of distinct patterns: asymmetric, rigid, spike, and/or box-like patterns (Figure S4). We developed a model based pattern-matching algorithm, HAYSTACK, in order to use these patterns to identify transcripts that change abundance over the day. HAYSTACK uses least-square linear regression for each transcript against all model cycling patterns with 24 possible phases (Figure S5). A series of statistical tests were used to identify the best-fit model, phase-of-expression, and to estimate a p-value and false-discovery rate (FDR) [3,4] for each gene. The estimation of cycling transcripts using HAYSTACK is greater than that with COSOPT (Table S1) [5]. The increased detection of cycling transcripts over COSOPT may result from the fact that the spike model detected 25-50% of cycling transcripts across all of the time courses (Figure 2D). Furthermore, the spike model identified more cycling transcripts under diurnal conditions, with the exception of temperature cycles alone, whereas the cosine model identified more cycling transcripts under circadian conditions (Figure 2D). These results are consistent with our hypothesis that diurnal and circadian expression have driven and smooth patterns, respectively.

Permutation-based false-discovery rate estimator to establish the HAYSTACK correlation threshold.

We used a permutation-based method to estimate the false-discovery rate (FDR) [3,4] for the correlations between each time course data series and the best matching HAYSTACK model. The data series for each Affymetrix probeset was permuted 1000 times, and for each permutation, we recorded the correlation between the permuted series and best HAYSTACK model for the real data. A *p*-value was estimated by dividing the number of times that the correlation of the permuted data series to the best model was greater than or equal to the correlation for the real data to the best model. The *p*-values were then imported into the Q-value package (http://faculty.washington.edu/jstorey/qvalue/) [3] with R (http://www.r-project.org/) to estimate FDRs. We selected cycling genes using a correlation cutoff of 0.8, which corresponds to a maximum FDR of 3.1% - 5.8% in different datasets. Separately, we used a perl script to estimate FDRs using the Benjamini & Hochberg method [4], which gave similar results.

**Genes called absent on the Affymetrix ATH1 Genechip**

The Affymetrix MAS5 software calls gene models ‘absent’, ‘missing’ or ‘present’ depending on how consistently the oligonucleotide probes covering a gene model behave in an experiment. Therefore, genes that are called present reflect probe sets that are statistically consistent across an experiment. This measure reflects both the performance of a probe set to estimate the transcript abundance of a specific gene, and whether the transcript is expressed in the tissue sampled in the microarray experiment. Gene models declared ‘absent’ or ‘missing’ by Affymetrix MAS5 algorithm at more than nine of twelve time points across a time course were removed from the HAYSTACK analysis (Table S3). Across all eleven arrays, between 23% and 35% of genes on the arrays were absent at all time points under a given condition, and approximately 50% of the genes were ‘present’ at every time point in any given time course. Of the genes that were always called absent across the time courses, there was significant overlap with genes always called absent in the AtGenExpress developmental series [6]. This result suggests that these probes do not accurately reflect the transcript abundance of the gene model they are predicted to measure, explaining why they are never called present despite diverse tissue types, developmental stages and genotypes.

**Phase calling using HAYSTACK**

A key aspect of diurnal and circadian regulation is the partitioning of specific biological activities to distinct times, or phases, over the day. When searching for cycling genes, models were computationally shifted by one-hour increments, modulating the waveforms to match our 4-hr sampling time (Figure S5). This allowed prediction of phase in 1-hr increments. Across the eleven time courses, transcripts were identified showing peak abundance at every phase over the day (Figure 5A, B). The phase of core clock genes is presented in Table S6, highlighting three important results of HAYSTACK phase prediction. First, phases are consistent with published microarray results (Table S6) [7]. Second, the phase of clock genes is approximately the same across conditions despite the fact that the time-courses were performed in multiple labs, using different conditions and genotypes (Table S6, Figure 1). Third, based on clock gene phase prediction, HAYSTACK is accurate within one hour. The best comparison is between the two LDHH conditions performed in two labs with different sampling strategies (LDHH_ST vs. LDHH_SM, Table S6). The accuracy of HAYSTACK in predicting phase was also independently verified during the promoter analysis (see Figure 8). It should also be noted that several so called ‘housekeeping’ genes such as ACTIN2 and TUBULIN4, used as references to normalize gene expression data, cycle under multiple conditions (Table S4). Table S5 provides a list of highly expressed genes that don’t cycle under any condition, which would be ideal to use as reference genes.

**ELEMENT**

The exhaustive enumerative methods we and others have developed estimate the probability of short DNA sequences, or ‘words’, occurring some number of times by comparing the count in a set of co-regulated sequences to an expected count based on random sampling or statistical modeling of a background distribution [8-11]. Earlier versions of our script [10] used a random sampling approach to model the background sequence and calculated z-scores for each 3-8 mer DNA word based on a comparison of the number of occurrences of that word in the upstream sequences of the co-expressed genes against a background distribution derived by random sampling of the upstream sequences of all genes represented on the microarray. The current version of our algorithm has been improved considerably, using a database of pre-calculated statistics for 3-8 mer words in the promoters of all genes represented on the Affymetrix ATH1 array to estimate the z-scores and p-values. The script also applies multiple hypothesis testing corrections [3,12] and binomial filters for each word ([http://element.cgrb.oregonstate.edu](http://element.cgrb.oregonstate.edu/)).

**Published time courses used in this study**

The LDHH_ST, LDHH_SM, LL_LDHH-SH, and LL_LDHH-AM time courses were previously described [13-16] (See Figure S3 and Table 1 for a description). In these published time courses, there are notable differences in sampling strategy from our time courses (Figure S3). The LDHH_ST time course was sampled every four hours in duplicate over only one day. The LDHH_SM time course was also sampled over one day, although some time points were collected more frequently than every 4-hrs. However, we only utilized time points taken at 4-hr intervals. The LL_LDHH-SH and LL_LDHH-AM time course was sampled on days 2 and 3 after release into continuous conditions compared to our time courses that were sampled on day one and two after release.

**Conservation of circadian networks across species**

To address the possibility of conservation across species of the circadian transcriptional networks we pursued a series of purely computational exercises to compare promoter architecture between *Arabidopsis*, rice and poplar, the three higher plant species for which high quality genome sequences exist [17-19]. The *Arabidopsis*, rice, and poplar predicted protein sequences representing annotated gene models were downloaded. All-versus-all reciprocal BLAST [20] was used to identify putative *Arabidopsis*-poplar and *Arabidopsis*-rice orthologs using the “mutual-best-blast-hit” criteria [21]. This analysis identified 12,730 and 10,040 putative *Arabidopsis*-poplar and *Arabidopsis*-rice orthologous pairs, respectively. Next, we used the annotated gene model coordinates to extract 500bp of putative regulatory DNA sequences upstream of the ATG of each rice or poplar putative ortholog. A first question is how much sequence homology exists between pairs of putative orthologous promoter sequences? To address this question we compared pairs of 500bp promoters between putative ortholog pairs using bl2seq (Figure S8). The resulting sequence matches were summarized into frequency distributions based on the length of matched sequence. This analysis indicated that most promoter segments conserved between orthologous *Arabidopsis*, poplar, and rice promoters are very short, with the vast majority of matches shorter than 10 bases, suggesting that there is very little long-range homology between the upstream regulatory regions of orthologs in these three species. In addition, a comparison of the genomic regions around a randomly chosen set of orthologs suggested that sequence conservation is largely limited to the coding region. Taken together, these observations are consistent with a model for promoter architecture in which short conserved islands of sequence occur in a background of diverged sequence, similar to what has been observed among yeast species [22].

Next, based on the assumption that the putative poplar and rice genes will cycle with the same phase as the *Arabidopsis* ortholog [23,24], we generated gene lists for each phase for poplar and rice under all the conditions tested. The 500bp upstream (from the ATG) sequences of the genes in the simulated rice and poplar phase bins were analyzed using ELEMENT, with background models derived from the promoters of all orthologs in each species, to identify over-represented words and generate z-score profiles. The resulting z-score profiles were then compared across species, identifying numerous examples of over-represented words displaying very similar z-score profiles and thus patterns of enrichment across phases in all three species for both known and potentially novel words (Figure 9). The observed conservation of z-score profiles across such great evolutionary distance is striking because the analysis is based on several assumptions, including correct identification of orthologs, the assumption that the putative orthologs actually cycle with the same phase as their *Arabidopsis* counterparts, and that the 500bp putative promoter regions used in the analyses represent true regulatory sequences.

**References**

1. Straume M (2004) DNA microarray time series analysis: automated statistical assessment of circadian rhythms in gene expression patterning. Methods Enzymol 383: 149-166.

2. Wijnen H, Naef F, Young M (2005) Molecular and statistical tools for circadian transcript profiling. Methods Enzymol: 341-365.

3. Storey JD, Tibshirani R (2003) Statistical significance for genomewide studies. PNAS 100: 9440-9445.

4. Benjamini Y, Hochberg Y (1995) Controlling the false discovery rate: a practical and powerful approach to multiple testing. JRoy Stat Soc B: 289 - 300.

5. Panda S, Antoch MP, Miller BH, Su AI, Schook AB, et al. (2002) Coordinated transcription of key pathways in the mouse by the circadian clock. Cell 109: 307-320.

6. Schmid M, Davison T, Henz S, Pape U, Demar M, et al. (2005) A gene expression map of *Arabidopsis thaliana* development. Nat Genet 37: 501-506.

7. Harmer SL, Hogenesch JB, Straume M, Chang H-S, Han B, et al. (2000) Orchestrated transcription of key pathways in *Arabidopsis* by the circadian clock. Science 290: 2110-2113.

8. Marino-Ramirez L, Spouge JL, Kanga GC, Landsman D (2004) Statistical analysis of over-represented words in human promoter sequences. Nucl Acids Res 32: 949-958.

9. Hudson M, Quail P (2003) Identification of promoter motifs involved in the network of phytochrome A-regulated gene expression by combined analysis of genomic sequence and microarray data. Plant Physiol 133: 1605-1616.

10. Nemhauser JL, Mockler TC, Chory J (2004) Interdependency of brassinosteroid and auxin signaling in *Arabidopsis*. PLoS Biology 2: e258.

11. van Helden J, Andre B, Collado-Vides J (1998) Extracting regulatory sites from the upstream region of yeast genes by computational analysis of oligonucleotide frequencies. J Mol Biol 281: 827-842.

12. Manly KF, Nettleton D, Hwang JTG (2004) Genomics, prior probability, and statistical tests of multiple hypotheses. Genome Res14: 997-1001.

13. Blasing O, Gibon Y, Gunther M, Hohne M, Morcuende R, et al. (2005) Sugars and circadian regulation make major contributions to the global regulation of diurnal gene expression in *Arabidopsis*. Plant Cell 17: 3257-3281.

14. Smith S, Fulton D, Chia T, Thorneycroft D, Chapple A, et al. (2004) Diurnal changes in the transcriptome encoding enzymes of starch metabolism provide evidence for both transcriptional and posttranscriptional regulation of starch metabolism in *Arabidopsis* leaves. Plant Physiol 136: 2687-2699.

15. Covington MF, Harmer SL (2007) The circadian clock regulates auxin signaling and responses in *Arabidopsis*. PLoS Biology 5: e222.

16. Edwards KD, Anderson PE, Hall A, Salathia NS, Locke JC, et al. (2006) *FLOWERING LOCUS C* mediates natural variation in the high-temperature response of the *Arabidopsis* circadian clock. Plant Cell 18: 639-650.

17. Tuskan GA, DiFazio S, Jansson S, Bohlmann J, Grigoriev I, et al. (2006) The genome of black cottonwood, *Populus trichocarpa* (Torr. & Gray). Science 313: 1596-1604.

18. Yu J, Hu S, Wang J, Wong GK-S, Li S, et al. (2002) A draft sequence of the rice genome (Oryza sativa L. ssp. indica). Science 296: 79-92.

19. Arabidopsis Genome Initiative (2000) Analysis of the genome sequence of the flowering plant *Arabidopsis thaliana*. Nature 408: 796-815.

20. Altschul S, Madden T, Schaffer A, Zhang J, Zhang Z, et al. (1997) Gapped BLAST and PSI-BLAST: a new generation of protein database search programs. Nucleic Acids Res 25: 3389 - 3402.

21. Tatusov RL, Koonin EV, Lipman DJ (1997) A genomic perspective on protein families. Science 278: 631-637.

22. Chin C-S, Chuang JH, Li H (2005) Genome-wide regulatory complexity in yeast promoters: Separation of functionally conserved and neutral sequence. Genome Res 15: 205-213.

23. Murakami M, Ashikari M, Miura K, Yamashino T, Mizuno T (2003) The evolutionarily conserved OsPRR quintet: rice pseudo-response regulators implicated in circadian rhythm. Plant Cell Physiol 44: 1229-1236.

24. Murakami M, Matsushika A, Ashikari M, Yamashino T, Mizuno T (2005) Circadian-associated rice pseudo response regulators (OsPRRs): insight into the control of flowering time. Biosci Biotechnol Biochem 69: 410-414.

**Supplemental Figures**

Figure S1. Daily interactions of temperature and light in nature by month.

Hourly temperature (°C, dotted line) and light (solar radiation, watts/m^2^, filled line) data were obtained for Princeton, Kentucky, USA from the Soil Climate Analysis Network (<http://www.wcc.nrcs.usda.gov/scan/>); SCAN site number: 2005; Latitude: 37° 06' N; Longitude: 87° 50' W; Elevation: 615 feet. Hourly data from April over three consecutive years were reformatted and averaged by hour over the month, and plotted over the 24-hour day. Data are averaged by month and day over three years. Daily relationship between temperature and light during an average day in April (A), October (B), July (C), and January (D).

Figure S2. Daily interactions of temperature and light in nature by latitude.

Hourly temperature (°C, dotted line) and solar radiation (watts/m^2^, filled line) data were obtained from the Soil Climate Analysis Network (wcc.nrcs.usda.gov/scan/). The data were analyzed as described in Figure S1. (A) Daily relationship between temperature and light during an average day in April. SCAN site number: 2010; Newton, Newton County, MS, USA; Latitude: 32° 20' N; Longitude: 89° 05' W; Elevation: 300 feet. Data average from April of 1999. (B) Daily relationship between temperature and light during an average day in April. SCAN site number: 2005; Princeton, Kentucky, USA; Latitude: 37° 06' N; Longitude: 87° 50' W; Elevation: 615 feet. Data average from April of 1999. (C) Daily relationship between temperature and light during an average day in April. SCAN site number: 2043; Mascoma River, Grafton County, NH, USA; Latitude: 43° 47' N, Longitude: 72° 02' W, Elevation: 14 feet. Data average from April of 2000.

Figure S3. Sampling strategy for diurnal and circadian time courses.

Light: white boxes represent the "lights on" conditions and black boxes represent "lights off". Temperature: blue boxes represent low temperatures (12°C) and white boxes represent high temperatures (22°C). Note, two replicates were sampled over one day for both LDHH_ST and LDHH_SM, and sampling began in the evening for the later time course. Also, samples were collected on days two and three starting at 26 hrs (CT2) after subjective dawn, as compared to all other time courses that that start at CT0. Grey boxes represent subjective night or cold during circadian time courses.

**Figure S4. Models used to identify diurnal and circadian regulated transcripts.**

(A) Rigid, (B) Spike, (C) Cosine, (D) Box2, (E) Box1, and (F) Asymetrix rigid model (asyrigid).

**Figure S5. Models shifted by one-hour increments to quantify time of transcript abundance.** All models were shifted in 1-hr increments causing a change in the model form and shape over the day to account changing waveforms caused by our four-hour sampling strategy. The models are presented in batches covering four-hour time intervals for presentation purposes.

(A) The spike model shifted to ZT00, ZT01, ZT02 and ZT03.

(B) The spike model shifted to ZT04, ZT05, ZT06 and ZT07.

(C) The spike model shifted to ZT08, ZT09, ZT10 and ZT11.

(D) The spike model shifted to ZT12, ZT13, ZT14 and ZT15.

(E) The spike model shifted to ZT16, ZT17, ZT18 and ZT19.

(F) The spike model shifted to ZT20, ZT21, ZT22 and ZT23.

**Figure S6. 2peak model identifies transcripts under long days**

(A) Number of transcripts called by the 2peak model across conditions.

(B) The expression pattern of PHOT1, which was called by the 2peak model under long day photocycles, under short day (black solid), long day (black dash) and LDHH_ST (grey).

**Figure S7. Flanking sequence distinguishes the G-box from the Morning Element.** All words identified in the cis-regulatory element analysis pipeline that overlap with the morning element (ME) were aligned. Two classes of elements emerged based on flanking sequence that share a CCAC core (yellow shading). The distinguishing sequence is highlighted for both ME (purple) or the Gbox (blue), and were summarized ccacAC and ccacGTG respectively. Graphing of the z-score profiles of ccacAC (blue) and cacGTG (red) predict that they have distinct temporal activity. ccacAC displays maximum overrepresentation 23 hours after dawn and cacGTG displays maximum overrepresentation 4 hours after dawn.

**Figure S8. Multiple PBX::LUC show rhythmic behavior under LDHH.**

(A) 10 independent T2 lines out of 10 display rhythmic behavior under LDHH (experiment TM185 for which average trace is displayed on Fig 8B).

(B) 4 independent T2 lines out of 4 display rhythmic behavior under LDHH (experiment TM186 for which average trace is displayed on Fig 8B).

(C) Z-score profile of the negative control element UNDER1 (GGACGTAC) under LLHC. The under1 element doesn’t any phase overrepresentation under any conditions tested.

(D) Bioluminescence of 5 independent T1 lines of UNDER1::LUC un LLHC.

(E) P-values derived from all the T2 progeny for the PBX::LUC analyzed in three independent experiment. Included is the P-value for the positive (EE-CCR2::LUC) and negative (UNDER1::LUC) controls.

**Figure S9. Only short DNA segments conserved between promoter regions of *Arabidopsis*, rice and poplar.** Promoter regions (500 bp) were aligned using BLASTN (bl2seq) between *Arabidopsis* and poplar (black) or *Arabidopsis* and rice (red).

**Figure S10. Circadian transcriptional network model.** The central feedback loops are an approximation of the circadian clock network in Arabidopsis. Timing is entrained by input signals such as photocycles and thermocycles. Rhythmic transcription factors act upon three important regulatory motif groups (the ME/G-box, GATA/EE, and the PBX/SBX/TBX) via transcription factors. Photosynthesis and protein synthesis/cell cycle are timed to occur in the morning and evening, respectively. Diurnal regulated both occurs in the morning while specifically clock regulated growth occurs in the evening.

**Supporting Tables**

**Table S1. Comparison of genes called rhythmic between HAYSTACK and COSOPT.**

**Table S2. All significant words identified in the promoter pipeline**.

**Table S3. Genes call absent by Affymetrix MAS5 algorithm.**

**Table S4. Phase of commonly used reference genes.**

**Table S5. Possible reference genes with good expression that do not cycle.**

**Table S6. Phase of known circadian clock genes in this study.**

**Table S7. Genes that cycle under all eleven diurnal and circadian conditions tested.**

**Table S8. Percent overlap between diurnal and circadian regulated genes.**

Percentage of genes captured by the condition at the top on the lower diagonal, and the percentage of genes captured by the condition on the left on the top diagonal. For instance, LLHC captures 70% of the genes in LL_LLHC (lower diagonal) and LL_LLHC captures 33% of the genes in LLHC (upper diagonal).

**Table S9. Transcripts called with the 2peak model across all eleven conditions.**
